# Performance Evaluation of Solar-Blind Gas-Filled Sensors to Detect Electrical Discharges for Low-Pressure Aircraft Applications

**DOI:** 10.3390/s22020492

**Published:** 2022-01-10

**Authors:** Jordi-Roger Riba, Manuel Moreno-Eguilaz, Maxence Boizieau, Tamerlan Ibrayemov

**Affiliations:** Campus Terrassa, Universitat Politècnica de Catalunya, Rambla Sant Nebridi 22, 08222 Terrassa, Spain; manuel.moreno.eguilaz@upc.edu (M.M.-E.); maxence.boizieau@reseau.eseo.fr (M.B.); ibrayemov.tamerlan@gmail.com (T.I.)

**Keywords:** aircraft power systems, low pressure, solar-blind sensors, ultraviolet radiation

## Abstract

Unpressurized aircraft circuits facilitate the initiation of electrical discharges in wiring systems, with consequent damage to related insulation materials and safety hazards, that can and have already caused severe incidents and accidents. Specific sensors and solutions must be developed to detect these types of faults at a very incipient stage, before further damage occurs. Electrical discharges in air generate the corona effect, which is characterized by emissions of bluish light, which are found in the ultraviolet (UV) and visible spectra. However, due to sunlight interference, the corona effect is very difficult to detect at the very initial stage, so the use of solar-blind sensors can be a possible solution. This work analyzes the feasibility of using inexpensive non-invasive solar-blind sensors in a range of pressures compatible with aircraft environments to detect the electrical discharges at a very incipient stage. Their behavior and sensitivity compared with other alternatives, i.e., an antenna sensor and a CMOS imaging sensor, is also assessed. Experimental results presented in this paper show that the analyzed solar-blind sensors can be applied for the on-line detection of electrical discharges in unpressurized aircraft environments at the very initial stage, thus facilitating and enabling the application of predictive maintenance strategies. They also offer the possibility to be combined with existing electrical protections to expand their capabilities and improve their sensitivity to detect very early discharges, thus allowing the timely identification of their occurrence.

## 1. Introduction

Due to the relentless development of a wide range of sensors and low-cost communication systems compatible with IoT applications, there is a growing effort to develop automated acquisition systems for detecting electrical discharges in power systems. In particular, aircraft power systems are more prone to electrical discharges due to the harsh environments and low-pressure conditions at which they are exposed. Recent and future developments in the area of more electric aircrafts (MEA) and all electric aircrafts (AEA) highlight the need to increase distribution voltage levels beyond 1 kV, to limit the current requirements and the weight of distribution wiring systems. However, operation at higher voltage levels exposes aircraft distribution systems to new challenges, such as an increased risk of electrical discharge occurrence, which is accentuated by the harsh aircraft environmental conditions, and specifically the low pressure, which significantly reduces the dielectric strength of air [1,2,3]. According to the well-known Paschen’s law, when reducing the ambient pressure, electrical discharges are triggered at lower voltage levels [4,5], thus increasing the risk of partial discharges, arcing and electric breakdown [6].

Flight altitudes of current commercial airliners lie in the 33,000–42,000 feet (10,000–12,800 m) range, and military aircrafts can fly above 50,000 feet (15,200 m), which approximately corresponds to 10 kPa. Therefore, electric and electronic systems placed in unpressurized areas of aircrafts have to withstand a wide pressure interval [7], which can range from 100 kPa (sea level) to around 10 kPa (worst condition).

Electrical discharges in aircraft electrical systems must be avoided, since their effects can lead to severe consequences, while tending to spread through electrical wires, producing a fire hazard [8,9], shutting down the affected systems with the consequent loss of functionality. Current jetliners can include hundreds of km of electrical wires, so fault detection is an issue, because wiring faults cause many in-flight fires, mission aborts [10,11], emergency aircraft landings and aborted takeoffs [12]. The aviation industry is continually developing and improving condition monitoring systems, although, even today, wiring issues frequently remain hidden. There is a pressing need to detect and assess the severity of insulation failures in existing aircraft wiring systems, as it is crucial to ensure stable, reliable and safe power system operation. However, the detection of arc tracking activity (the formation of a conducting path along the surface of cable insulation due to chemical and thermal decomposition or erosion of the insulation material) in the very early stage is an unsolved problem. Because of the low fault current produced, it is below the sensitivity of current circuit breakers, so they are only able to trip when the problem is in an advanced stage, and a certain level of damage has been produced. Arc tracking activity releases energy and generates different measurable effects [13], including radio waves, sound, visible and ultraviolet light, heat or chemicals such as ozone, NOx and nitric acid, among others [14]. Arc tracking activity tends to pyrolyze the insulation materials surrounding the core of the wire, thus producing a partially conductive path that promotes discontinuous arcing activity of low magnitude that overheats the insulation [15], thus producing more damage. Therefore, the low energy level associated with arc tracking activity in the very early stage makes its detection difficult [16].

Arc tracking often produces arcing in small air gaps, which in turn generates corona activity in the most stressed region [17,18]. Corona is typically detected by means of complex devices including UHF sensors [19], radio interference voltage and partial discharge detectors [20], spectrophotometers [21] or noise meters [22], among others. Corona activity can also be detected using visible and ultraviolet (UV) optical sensors [23], so these sensing methods offer the possibility to detect electrical discharges in the incipient state [2] using reduced-size and low-cost sensors. Visible/UV imaging can detect visible and UV emissions produced during the electrical discharge process, while allowing the determination of the discharge points [24]. However, sunlight also contains visible and UV wavelengths, thus interfering with the measurements of visible and UV sensors.

Solar radiation is mostly optical radiation, i.e., radiant energy emitted within a broad interval of the electromagnetic spectrum. It includes ultraviolet (UV), visible light and infrared radiation, although both ionizing radiation of shorter wavelength and microwaves and radiofrequency with longer wavelengths are also present. UV radiation falls in the 100–400 nm range, and it can be subdivided into UVC (100–280 nm), UVB (280–315 nm) and UVA (315–400 nm). UVC and most UVB extraterrestrial radiation is absorbed by stratospheric ozone. Optical sensors that are only sensitive to UVC radiation, i.e., sensitive below the 280 nm interval, are known as solar-blind sensors [25], because they can only detect electromagnetic radiation with wavelengths shorter than those of the solar radiation once it has penetrated the atmosphere of the Earth. Therefore, solar-blind sensors do not produce any measurable signal when exposed to ordinary outdoor lighting. However, the concept of solar blindness in space changes, because of the lack of an atmosphere between the Sun and the sensor [26].

Solar-blind sensors emerge as a plausible alternative to avoid sunlight interference, so this possibility is analyzed in this paper. To this end, laboratory experiments comparing the sensitivity of solar-blind UV sensors against the sensitivity of an antenna sensor and a visible/UV CMOS imaging sensor are conducted at a wide range of pressures covering most aircraft applications, this being a contribution of this work. Results presented here also show the key role of pressure in the corona extinction voltage of a wire electrode, thus providing valuable experimental data for designing wiring insulation systems for electrical circuits found in MEA and AEA aircrafts. These applications require extensive testing for the accurate prediction of the corona extinction voltage under low-pressure conditions. The sensing approach proposed in this work seeks to identify discharge activity in the very initial stage, well before the development of major faults.

The paper is organized as follows. Section 2 describes the experimental setup and the electrodes and sensors used to detect the electrical discharges in the very early stage. Section 3 presents and comments on the experimental results, and, finally, Section 4 concludes the paper.

## 2. Experimental Setup

This section describes the experimental setup, including the electrodes and apparatus used to generate the electrical discharges, the low-pressure chamber where the tests were carried out, as well as the sensors to detect the UV light at the very initial stage of the discharge.

### 2.1. The Analyzed Sensors

**Figure 1:**
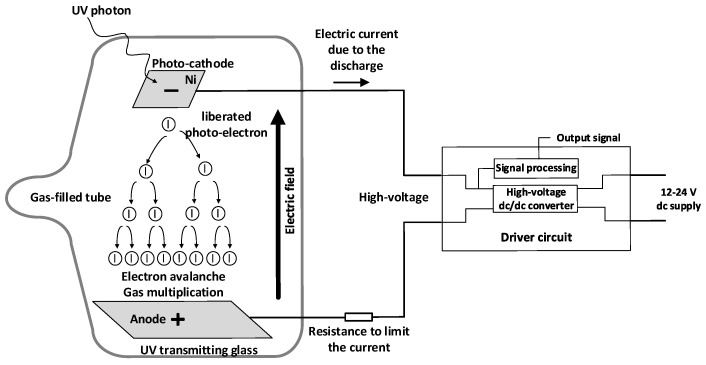
Schematics of a gas-filled tube to multiply the liberated photo-electrons by the incident UV photons and the driver circuit.

The UVTRON is a binary (on/off) sensor sensitive to ultraviolet. It exhibits a fast and very narrow spectral response in the 185–260 nm interval, thus being solar-blind and insensitive to visible light. Since electrical discharges typically emit ultraviolet light, this sensor is well suited to their detection. Its response is based on the photoelectric effect. When an ultraviolet photon of adequate wavelength strikes the nickel electrode (photocathode or negative electrode), an electron is released. The UVTRON consists of a gas-filled tube, where an electron avalanche multiplication phenomenon takes place between two metallic electrodes, the photo-cathode and the anode. The two electrodes are subjected to a high-voltage difference to sustain the avalanche. The avalanche is initiated or triggered when incident UV photons strike the photo-cathode and liberate electrons, known as photo-electrons. Due to the high voltage applied between the two electrodes and the original photo-electrons liberated inside the gas-filled tube by the incident UV photons, a gas multiplication or electron avalanche occurs, in which each subsequent collision liberates a new electron, with the consequent multiplicative effect, thus sustaining the avalanche. These free electrons accelerate towards the anode, guided by the strong electric field, colliding with gas molecules, ionizing such molecules and producing more electrons that are accelerated by the electric field. They collide with other gas molecules, thus producing more ionization and generating more electrons. This gas multiplication process is repeated until the electrons finally reach the anode. This type of electrical discharge is also known as Townsend discharge. Figure 1 details the structure of the sensor and the avalanche process.

Table 1 summarizes the main characteristics of the Hamamatsu gas-filled tube solar-blind sensors analyzed in this paper.

To maximize the performance of each sensor, a suitable driver circuit is required. To this end, the commercial C10807 and C10423 driver circuits were used jointly with the R9454 and R9533 sensors, respectively, since they can be supplied at a low voltage in the 12–24 V range, while integrating a high-voltage dc/dc converter and a signal processing circuit. Thus, the sensors can be operated by applying a low voltage through the drivers, while the signal processing circuit allows for a reduction in background noise.

To assess the performance of the UVTRON sensors, their sensitivity was compared against that of two other sensors, such as a low-cost back-illuminated CMOS imaging sensor and a loop antenna, which have been tested in previous works [24].

The first corona detection method detects the visual corona using an imaging sensor (48 Mp IMX586 CMOS sensor from Sony, Tokyo, Japan) sensitive to both visible and UV wavelengths [27]. However, this sensor is not solar-blind and only partially sensitive to the UV spectrum, although, due to the images generated, it allows the corona discharge points to be localized.

The second sensor is a single-loop antenna (diameter = 95 mm, made of enameled copper wire with diameter = 1.2 mm, approximate cutting frequency of 20 MHz). This sensor presents several advantages, such as low cost, light weight, reduced dimensions and very high sensitivity. Due to its superior sensitivity, this is the reference sensor in this paper, although it cannot be used in aerospace applications due to the great impact of electromagnetic noise typical of such applications on its response. The signal provided by the antenna sensor was acquired by using an isolated digital oscilloscope (RTH1004, Rohde & Schwarz, Munich, Germany, 5 GSa/s, 0–1000 V), which was connected to two passive voltage probes (RT-ZI10, Rohde & Schwarz, Munich, Germany, 1 kV, 500 MHz, 10:1).

### 2.2. The Wire Electrodes

This section describes the wire electrodes used to assess the sensitivity of the sensors analyzed in this work. Wire electrodes were analyzed since arc tracking frequently occurs between adjacent wires in aircraft circuits, this being a relevant electrode geometry to generate early-stage discharges. Moreover, standard methods to assess the resistance to arc tracking in wire electrodes are well described in the technical literature [28,29]. To facilitate corona activity, the core of one of the conductors was connected to the high-voltage terminal of the high-voltage generator, whereas the other was connected to the ground.

**Figure 2:**
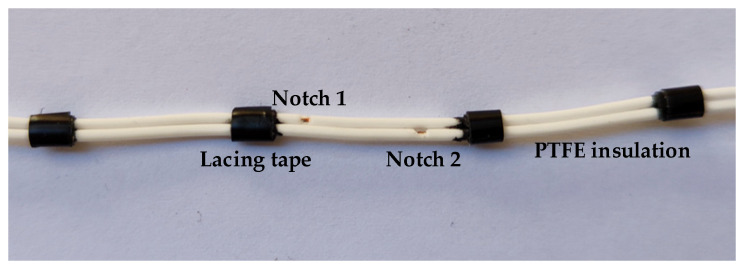
Polytetrafluoroethylene (PTFE)-insulated wire electrodes used to test the different sensors. The width of the notches is 1 mm and the distance between notches is 10 mm.

The wire electrodes were configured and artificially damaged according to the description found in the EN 3475-603:2018 standard [28], as shown in Figure 2.

Table 2 summarizes the properties of the analyzed PTFE-insulated wires.

### 2.3. The Low-Pressure Chamber

As explained, the pressure range in aircraft applications lies in the 10–100 kPa range. Therefore, experiments to simulate the behavior of the electrical discharges under these constraints must be performed inside a low-pressure chamber. A cylindrical stainless-steel low-pressure chamber was used for this purpose, with an inner diameter of 260 mm and a height of 375 mm. It included a methacrylate lid sealed with an elastic gasket to prevent the entry of air from the outside, thus allowing the wireless CMOS sensor to transmit photographs to an external computer. It also contained a vacuum-tight access port for the high-voltage cable and another one for the wires supplying the solar-blind gas-filled tube sensors. A vacuum pump was used to regulate the pressure, while it was measured by means of an analogic manometer. Experimental measurements were performed at 25 °C and humidity was limited below 25%.

During the tests, the methacrylate lid was covered with an opaque material to prevent sunlight from entering and interfering with the CMOS imaging sensor measurements.

### 2.4. The Programmable High-Voltage Source

Since the corona extinction voltage (CEV) value depends on the applied pressure, and the tests were performed under variable pressure conditions, a power supply capable of generating a variable high-voltage output was required. To this end, a low-voltage programmable power supply (0–300 V, ±0.1 V, SP300VAC600W, APM Technologies, Dongguan, China) was connected to a single-phase step-up high-voltage transformer (36 kV, VKPE-36, Laboratorio Electrotécnico, Barcelona, Spain), whereas the output frequency of the power supply was settled to 400 Hz, which is the typical frequency in aircraft applications. The voltage was regulated to determine the CEV value at each pressure.

**Figure 3:**
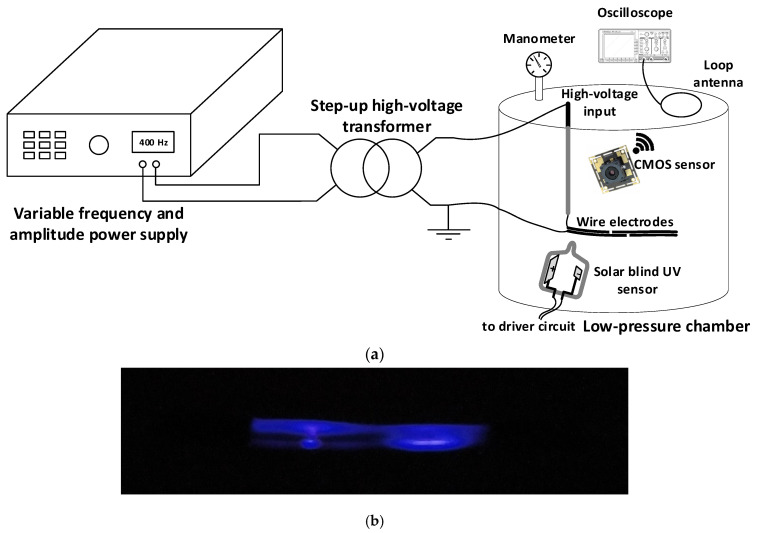
(**a**) Experimental setup sketch including the used instrumentation. The distance between the gas-filled sensors and the wire electrode is approximately 50 mm, whereas the distance between the CMOS image sensor and the wire is approximately 90 mm; (**b**) photograph of an early-stage discharge at 10 kPa when applying 700 V_RMS_ and 400 Hz, appearing in the surroundings of the notch sites.

Figure 3a details the experimental setup used in this paper to determine the CEV values of the wire electrodes under varying pressure conditions.

### 2.5. The Corona Extinction Voltage (CEV) Value

The data presented in this work are to determine the lowest voltage level at which corona activity is produced, so the CEV value is used. The corona tests carried out in this work were performed using the electrodes described in Section 2.2. To determine the CEV value at each pressure level, the voltage was initially set to 0 kV and progressively raised until corona activity was detected, this voltage corresponding to the corona inception voltage (CIV). Then, the voltage was further raised by around 10% and next progressively reduced until total extinction of the corona effect, the last point where corona activity can be measured being the CEV value. The CEV value corresponds to the lowest voltage at which corona activity can be measured.

It is noted that UVTRON sensors produce an on/off output signal whose frequency increases with the discharge level, thus making it possible to quantify the evolution of the discharge, this information being useful to develop predictive maintenance strategies. The response of these sensors can be combined with existing electrical protections after some modifications. When the sensor detects the presence of an initial discharge, an alarm signal can be sent to the protection, which in turn could send the alarm signal to an external computer, which could decide if further actions are required.

The cost of the UVTRON sensor and the driver circuit is around 20 to 50 times less than that of PD detectors, RIV detectors or UV cameras.

## 3. Experimental Results

This section describes the results attained with the different sensors to assess the sensitivity of the solar-blind UV-sensitive gas-filled sensors. The results presented in this section are based on the electrodes detailed in Section 2.2, by applying the procedure to detect the CEV value explained in Section 2.5.

### 3.1. Initial Experimental Tests with the Solar-Blind Sensors

To determine the suitability of using the UVTRON sensors to detect electrical discharges, different initial tests were performed under regular atmospheric pressure conditions.

The first test consisted of comparing the behavior of the solar-blind sensors under sunlight conditions and under total darkness, resulting in the same sensitivity for the UVTRON sensors under both conditions, thus proving that sunlight does not interfere their measurements.

The second test consisted of measuring the detection sensitivity of the sensors placed at a distance of 0.5 m from the discharge point (needle-plane gap in this case). This distance was selected because it was sufficient for the purpose of this study. The results attained are sown in Table 3.

The results in Table 3 show very similar detection limits of the UVTRON sensors compared to that of the CMOS imaging sensor, where, in a previous work [23], it was shown that the CMOS imaging sensor has almost the same sensitivity as one of the most sensitive and expensive sensors, a conventional PD (partial discharge) detector.

**Figure 4:**
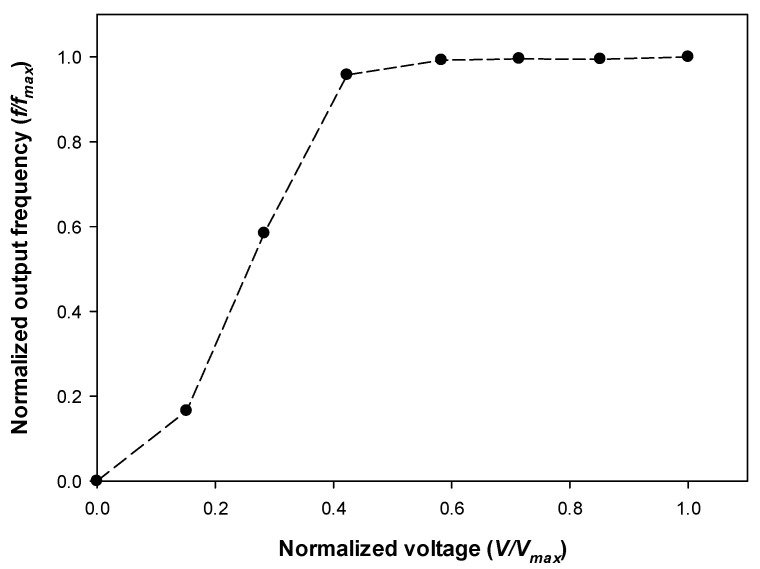
Normalized frequency response of the UVTRON R9454 sensor.

Finally, a third test was carried out for determining the frequency response of the sensors from below the CEV value (they produce a response of 0 Hz) to saturation (maximum frequency, *f_max_*). The results attained are shown in Figure 4.

The results in Figure 4 show the dependency of the output frequency of the sensors with the applied voltage, thus making it possible to quantify the intensity of the discharge, this information being useful to develop predictive maintenance strategies.

### 3.2. Experimental Tests with the Wire Electrodes Inside the Low-Pressure Chamber

This section deals with the wire electrodes, which were used to assess the sensitivity of the sensors analyzed in this work.

**Figure 5:**
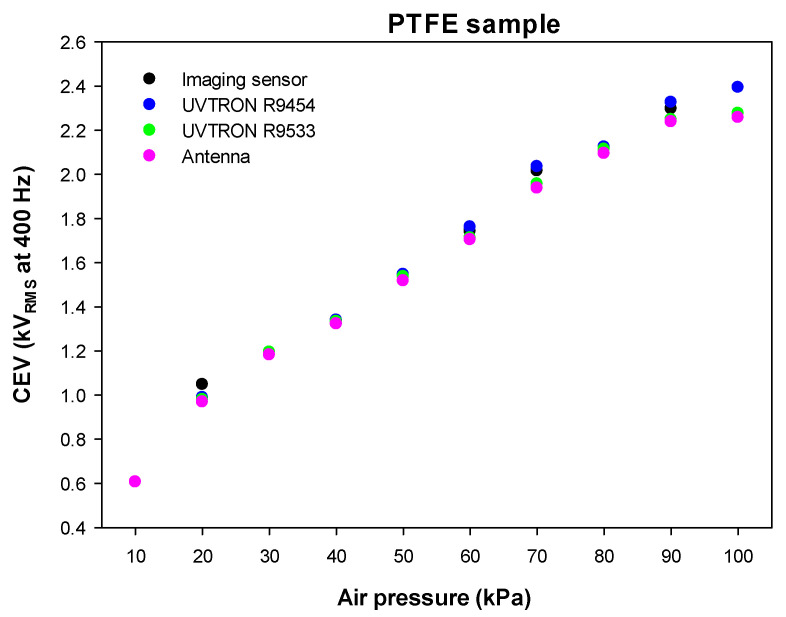
Results attained with the different sensors in the 10–100 kPa range covering the pressure range found in aeronautic applications using the PTFE-insulated wire electrodes depicted in Figure 2.

Figure 5 summarizes the results obtained in the low-pressure chamber using the antenna, CMOS imaging sensor and the two solar-blind gas-filled sensors within the pressure range covering the 10–100 kPa interval in steps of 10 kPa.

The results presented in Figure 5 clearly show a similar trend for the four analyzed sensors. A close examination suggests that, in most cases, the antenna is the most sensitive one, because it detects the very early corona activity at the lowest CEV values, followed by the UVTRON R9533 sensor, which, under the conditions of the tests, is the second most sensitive sensor. As already explained, although the antenna is the most sensitive method to detect the electrical discharges in the very early stage, this sensing method is strongly affected by electromagnetic noise, so it is difficult to apply in real environments.

For a better interpretation of the results presented in Figure 5, Table 4 shows the relative differences in the CEV values of the different sensors compared to the antenna, the most sensitive sensor, which is taken as the reference sensing method.

The results summarized in Table 4 show a great similitude among the CEV values determined by the different sensors. Compared to the antenna sensor, the most similar results are attained by the UVTRON R9533, exhibiting a mean difference with respect to the antenna of 0.79% (minimum and maximum differences of 0.00% and 1.35%, respectively), followed by the CMOS imaging sensor, with a mean difference of 2.60% (minimum and maximum differences of 0.00% and 8.15%, respectively).

## 4. Conclusions

This paper has analyzed the behavior and sensitivity of two solar-blind sensors in the 10–100 kPa pressure interval, which accounts for the pressure range found in unpressurized aircraft circuits. The analyzed solar-blind sensors are sensitive within the 185–260 nm ultraviolet spectral range. Their sensitivity has been contrasted with that of an antenna sensor and a CMOS imaging sensor, since, in previous works, the good performance of the two last sensors has been proven. A wire electrode has been used to assess the performance of the different sensors. Wiring systems in aircraft applications tend to produce discharges, and standard procedures to assess the resistance to arc tracking of wire insulation materials are found in the technical literature. Experimental results presented in this work clearly show the feasibility and accuracy of using solar-blind UV sensors to detect electrical discharges at the very incipient stage in low-pressure environments found in aircraft applications, well before irreversible damage in wiring systems is produced. The analyzed sensors have appealing features because they are inexpensive, allow non-invasive measurements, are suitable for on-line monitoring, are insensitive to sunlight interferences and have reduced dimensions and low power consumption. The use of these sensors facilitates the application of predictive maintenance plans while offering the possibility to be combined with existing electrical protections to expand their capabilities, thus allowing timely fault identification and a fast response.

## Figures and Tables

**Table 1:** Hamamatsu UVTRON sensors analyzed in this work.

Parameters	R9454	R9533
Manufacturer	Hamamatsu	Hamamatsu
UV range	185–260 nm	185–260 nm
DC supply voltage	400 ± 25 V	350 ± 25 V
Peak senssitivity	200 nm	200 nm
Sensitivity range > 10%	185–230 nm	185–230 nm
Maximum peak current	30 mA	30 mA
Estimated life	25,000 h	25,000 h
Operation temperature	−40 to 125 °C	−40 to 125 °C
Weight	1.5 g	2.5 g

**Table 2:** Properties of the analyzed PTFE-insulated wires.

Properties	Values/Description
Manufacturer	AlphaWire
Size	AWG 24 (7/32)
Applicable standards	AWM/STYLE 1213; MIL-W-16878/4 (Type E)
Insulation material	PTFE
Outer diameter	1.12 mm
Insulation thickness	0.25 mm
Temperature range	−60 to 200 °C
Voltage rating	600 VRMS

**Table 3:** Detection limit (CEV value) of the UVTRON sensors at a distance of 0.5 m from the discharge point.

Voltage (kV)	Imaging Sensor	R9454	R9533
8.0	Detection limit	≈0.1 Hz	No detection
8.5	Detection	1.5–1.75 Hz	≈0.1 Hz
9.0	Detection	3.0–3.5 Hz	0.25–0.5 Hz
10.0	Detection	4.0–5.0 Hz	≈1 Hz

**Table 4:** CEV values percentage difference (%) of the different sensors with respect to the antenna (most sensitive sensor) using PTFE-insulated wire electrodes.

Pressure (kPa)	Imaging Sensor	UVTRON R9454	UVTRON R9533
10	0.00	0.00	0.00
20	−8.15	−2.06	−1.14
30	−1.01	−1.01	−1.01
40	−1.28	−1.28	−0.76
50	−1.91	−1.91	−1.25
60	−2.29	−3.40	−0.53
70	−4.02	−5.01	−0.98
80	−1.38	−1.38	−0.91
90	−2.59	−3.93	−0.45
100	−0.84	−6.02	−0.84
Average	−2.35	−2.60	−0.79

## Data Availability

Not applicable.
